# Ocular Syphilis Revisited: A Review of Cases and Evidence From the Literature

**DOI:** 10.7759/cureus.88926

**Published:** 2025-07-28

**Authors:** Mahmoud Eissa, Dimitrios Kalogeropoulos, Farid Afshar, Nigel Hall, Najiha Rahman, Andrew J Lotery

**Affiliations:** 1 Ophthalmology, Mountainhall Treatment Centre, Dumfries, GBR; 2 Ophthalmology, University of Ioannina, Ioannina, GRC; 3 Ophthalmology, University Hospital Southampton National Health Service (NHS) Foundation Trust, Southampton, GBR

**Keywords:** eye infection, inflammation, masquerade, ocular syphilis, uveitis

## Abstract

Aim: This case series seeks to share our experience with ocular syphilis by reporting the clinical findings and management of nine diagnosed cases.

Materials and methods: This retrospective case series comprises nine patients who presented to the Ophthalmology Department of Southampton General Hospital between 2013 and 2022. The series provides a comprehensive report on patients diagnosed with ocular syphilis, detailing the severity of the condition, associated symptoms, and the treatments administered.

Results: All cases in the study were diagnosed with syphilis based on ocular findings. The patients exhibited a wide range of visual acuity, from normal to as low as light perception vision, with corresponding posterior segment results and varied intraocular pressure data. Some patients had recently been diagnosed with co-infection involving the human immunodeficiency virus. Except for one patient who underwent intravenous ceftriaxone treatment, the majority received systemic therapy with intravenous benzylpenicillin. In four cases, prednisolone was administered in conjunction with the systemic treatment. For topical therapy, three patients were provided with cycloplegics and topical steroids to alleviate discomfort and prevent iris adhesions.

Conclusions: Diagnosing ocular syphilis presents challenges, which emphasizes the value of excluding syphilis infection in patients exhibiting uveitis, optic nerve neuritis or atrophy, acute muscular palsy, or visual loss. Ocular syphilis warrants consideration during diagnosis, necessitates treatment akin to neurosyphilis, and mandates testing for other immunodeficiency diseases. Inadequate management may lead to persistent vision impairment.

## Introduction

The infectious disease syphilis is brought on by the spirochete *Treponema pallidum* (*T. pallidum*). Small wounds on the skin, especially on mucous membranes during intercourse, can transmit the disease, as can congenital transmission in utero, which can happen through the placenta or, less frequently, by coming into contact with an active genital lesion during delivery [1]. An estimated 12 million new cases of syphilis are reported annually, with developing nations accounting for the great bulk of these instances [2]. According to animal studies, *T. pallidum* organisms spread throughout the body within hours after injection, initially appearing in lymph nodes [3,4].

Ocular symptoms of syphilis may manifest at the primary, secondary, or tertiary stages of the disease. The eye structures susceptible to syphilitic involvement encompass the optic nerve, pupillomotor pathways, cranial nerves, conjunctiva, sclera, cornea, lens, uveal tract, retina, and retinal vasculature [3]. Ocular syphilis typically manifests in its advanced stages, featuring symptoms like eyelid chancres, glaucoma, cataracts, salt-and-pepper fundus appearance, chorioretinitis, and interstitial keratitis. While presentations may vary, delayed treatment poses the risk of permanent vision loss and other associated complications [4].

Given its wide range of clinical manifestations and its ability to mimic various other conditions, syphilis is commonly referred to as "the great imitator." As a result, maintaining a heightened level of suspicion is crucial for the accurate diagnosis of ocular illnesses associated with syphilis [3,5]. Identifying relevant indicators during the history-taking and physical examination, such as a history of multiple sexual partners, HIV infection, the presence of a saddle nose deformity, painless chancres, or a maculopapular rash affecting the palms and soles, can enhance the accuracy of diagnosing syphilis. The diagnostic approach involved both treponemal and non-treponemal tests, aligning with the prevailing consensus among authors who advocate the concurrent use of these diagnostic tools in neuro-ophthalmology for syphilis diagnosis [6]. This study aims to present the diagnostic and therapeutic considerations in nine cases of patients with ophthalmic syphilis.

## Materials and methods

This case series endeavours to share our insights into ocular syphilis by detailing the clinical findings and management of nine confirmed cases observed at the Ophthalmology department of Southampton General Hospital from 2013 to 2022, complemented by a comprehensive review of the existing literature on the condition. The patients included in this series exhibit a spectrum of severity, and their symptoms are outlined in Table 1. Additionally, Table 2 provides an overview of the treatments administered and the final ocular measurements recorded.

**Table 1:** Clinical presentations of nine ocular syphilis cases BE: both eyes, CME: cystoid macular edema, DM: descment membrane, F: female, FA: fluorescein angiography, FFA: fundus fluorescein angiography, ICGA: indocyanine green angiography, IOP: intraocular pressure, M: male, LE: left eye, RE; right eye, N: not applicable, SPKS: superficial punctate keratitis, VA: visual acuity, MRI: magnetic resonance imaging, US: ultrasound

No.	Demographics	Symptoms	Diagnosis	Uveitis	VA	IOP	Retinal imaging
1	25 yrs., F	White dots affect the vision in LE	Bilateral, ocular syphilis	Optic disc swelling	6/7.5 and 6/9	N	Optic disc oedema and swelling with cotton wool spots and exudation RE > LE with RE CME
2	65 yrs., M	Blurred vision + VI nerve palsy, initially diagnosed with sarcoidosis - mediastinoscopy biopsy of lymph node	Bilateral, syphilitic panuveitis	Vitritis LE > RE	6/9.5	15 BE	Bilateral panuveitis RE: hyperreflective area superior to fovea LE: area of thinning of inner retinal layers inferotemporal to fovea (both present in 2014) and right peripapillary hemorrhage
3	52 yrs., M	Symptoms of meningitis + continuous buzzing in his ears and muffled hearing in the left ear more than the right + blurred vision	Bilateral, neurosyphilis	Optic disc swelling, mild vitritis	6/6 BE	N	Brain imaging (MRI) shows a slight prominence of ventricles, US abdomen shows an enlarged spleen, and an audiogram: sensorineural high-frequency hearing loss (bilaterally)
4	26 yrs., M	extensive floaters and cobwebs in BE with tunnel vision in LE, and the eyes are throbbing	Bilateral, ocular syphilis	Intermediate (mild Vitritis and snowbanks)	HM	11 T 12	RE macula drusen
5	50 yrs., M	Blurred vision and red, painful, watery eyes	Bilateral, neurosyphilis	Posterior	6/15 and 6/24	N	RE with areas of atrophy and inferiorly round well-described areas of atrophy, vessel attenuation BE, generalised atrophy at OCT, FAF shows patches of atrophy BE
6	57 yrs., M	Acute painless visual loss for 6 days	LE, ocular syphilis	LE retinitis as subretinal white lesions	6/30 and 6/60	18t14	Slight loss of the is/os layer inferotemporal to the fovea and extending superiorly like an arc, the left color photo shows some slight pale area centrally around the fovea, corresponding to loss and disturbance of the is/os in this area with some spiky projections from the RPE. AF right superior hypoautofluor, ffa/icga left eye showed late leaks around the terminal vascular branches
7	75 yrs., M	Blurred vision, red, painful, watery eyes, and photophobia	Bilateral/left amblyopia, ocular syphilis	Panuveitis	4/60 RE 3/60 LE	10 BE	Changes of a "salt and pepper" type with evidence of persisting perivenous sheathing, particularly in the retinal periphery. This was confirmed by widespread mottled hyper and hyperfluorescence, venous leakage, and signs of ischemia on fluorescein angiography of the right eye. Fundus examination of the left eye showed marked retinal whitening and hemorrhages centred around the posterior pole
8	32 yrs., M	Blurred vision + 10-day history of sudden onset central scotoma in the LE with photopsia, first diagnosed as Krill's disease	Bilateral, ocular syphilis	Bilateral syphilitic chorioretinitis (L > R)	6/12 and 6/24	14/15	FFA: generalized vasculitis + disc leakage
9	46 yrs., M	Redness, pain, photophobia, blurred vision	RE, neurosyphilis	Anterior uveitis (non-granulomatous); corneal SPKs, DM folds, perilimbal injection	6/60 and 6/6	8 in BE	Nil in Heidelberg OCT, FFA

**Table 2:** Treatment options and the final ocular measurements BE: both eyes, F: female, IOP: intraocular pressure, M: male, LE: left eye, NA: not applicable, PRP: panretinal photocoagulation, VA: visual acuity

No.	Demographics	Topical treatment	Systematic treatment	Final VA	Final IOP
1	25 yrs., F	N/A	IV benzylpenicillin and oral pred	6/7.5 BE	N/A
2	65 yrs., M	Dexamethasone 0.1% and cyclopentolate 1% eye drops	Started on topical treatment and referred again to infectious diseases	UA6/6 and 6/9	7 T 6
3	52 yrs., M	N/A	IV benzylpenicillin 14 days switched to IM procaine penicillin from 4/4 to 9/4 and one dose of oral steroids	6/6 in BE	N/A
4	26 yrs., M	Dexamethasone 0.1%, brinzolamide/timolol combined eye drops	1g methylpred before diagnosis, then 2/52 IV benzylpenicillin and 3/7 oral pred	6/7.5 and 6/6	14 T 12
5	50 yrs., M	N/A	N/A	6/6 + 6/9	6T7
6	57 yrs., M	N/A	IV ceftriaxone + oral prednisolone 60 mg	6/7.5 BE	15 t 16
7	75 yrs., M	Sectoral PRP (LE)	N/A	3/60/hand motion	20 t 16
8	32 yrs., M	N/A	IV penicillin + 3/7 500 mg methylprednisolone	6/7.5 BE	15 T 16
9	46 yrs., M	Dexamethasone 0.1% and cyclopentolate 1% eye drops	IV benzylpenicillin 10 days (initially IV ceftriaxone 5 days)	UA 6/24, PH 6/18 and fellow eye 6/7.5	14 T 15

## Results

Case reports

Case 1

**Figure 1:**
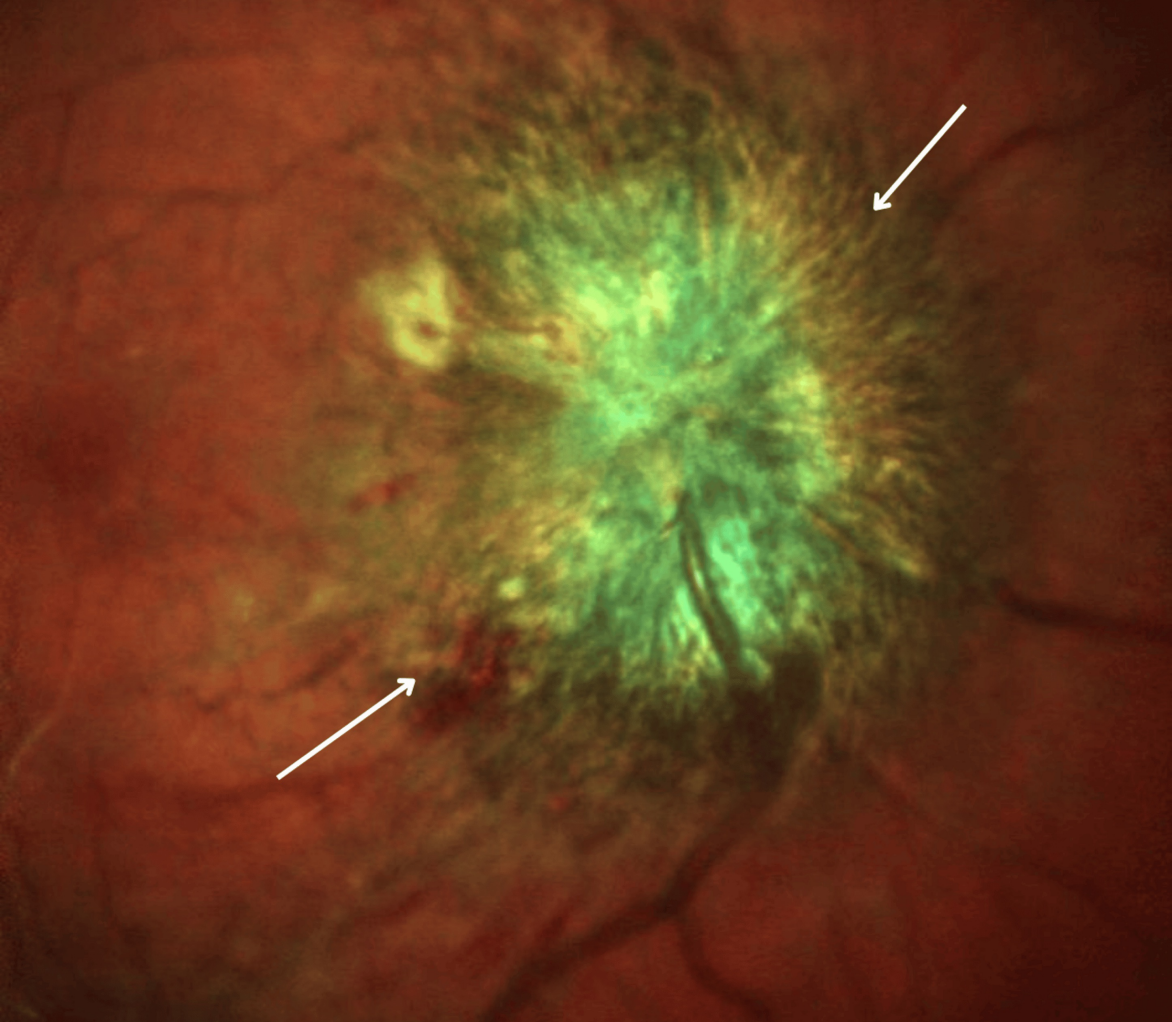
SLO wide-field image showing right optic disc swelling secondary to optic neuritis/perineruitis SLO wide-field image of the using Heidelberg. The image highlights optic disc swelling (arrows), consistent with optic neuritis/perineuritis. SLO: scanning laser ophthalmoscopy

A 25-year-old female presented with visual disturbances characterized by white dots in her left eye, alongside optic disc swelling observed upon examination. Retinal imaging revealed edema and swelling in the optic disc, as shown in Figure 1, with cotton-wool spots and exudation predominantly evident in her right eye, along with cystoid macular edema. Referred by the neurologist, she received a diagnosis of bilateral ocular syphilis, with visual acuity (VA) measured at 20/25 in the left eye and 20/32 in the right eye, and intraocular pressure (IOP) within normal limits. Intermittent headaches were noted as her systemic symptoms, and laboratory tests indicated a Venereal Disease Research Laboratory test (VDRL) titer of 1:4 and a *T. pallidum* particle agglutination assay (TPPA) titer of 1:1280. Treatment involved intravenous benzylpenicillin (3 million units every four hours for 14 days) and oral prednisolone (started on 40 mg per day, followed by gradual tapering) as systemic interventions. Subsequently, she was referred to the neurology department to rule out idiopathic intracranial hypertension. The final examination in 2021 revealed a VA of 20/25 for both eyes.

Case 2

**Figure 2:**
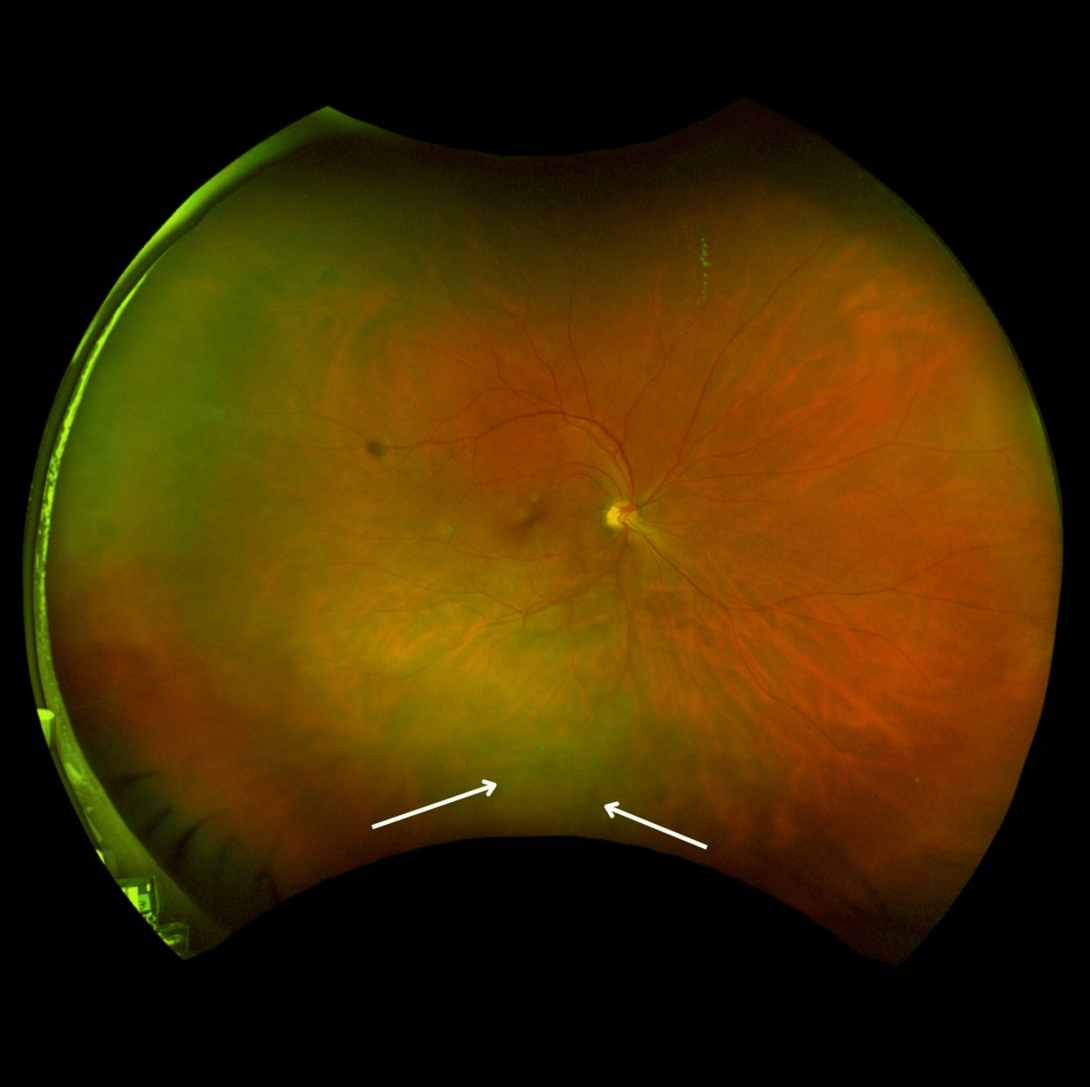
SLO wide-field image showing right inferior exudates with vitreous cells and with retinitis Multicolor SLO photograph of the right eye, demonstrating right inferior exudates with retinitis (arrows). SLO: scanning laser ophthalmoscopy

**Figure 3:**
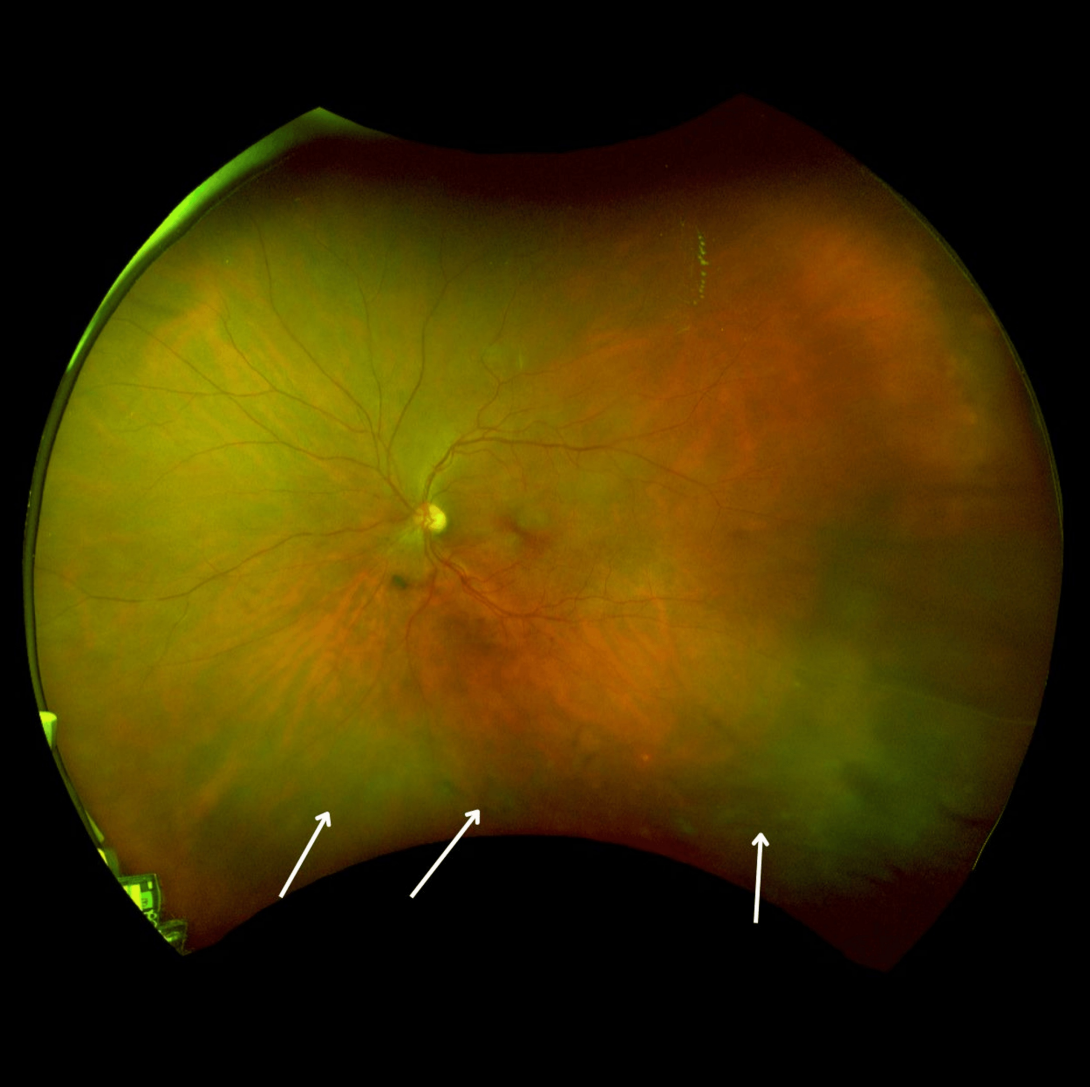
SLO wide-field image showing left eye with inferior exudates with vitreous cells with retinitis Multicolor SLO fundus photograph of the left eye. The image demonstrates inferior exudates with vitreous cells and retinitis (arrows). SLO: scanning laser ophthalmoscopy

A 65-year-old man presented for his initial examination with complaints of blurred vision and sixth nerve palsy. His medical history included pulmonary sarcoidosis, a prior diagnosis of prostate cancer, cardiac valve replacement, and a history of syphilis four decades ago. Upon examination, vitritis was noted in the left eye, as seen in Figures 2-3, with VA measuring 20/32 and IOP recorded at 15 mmHg for both eyes. The diagnosis revealed bilateral syphilitic panuveitis. Retinal imaging revealed a hyperreflective area superior to the fovea in the right eye and thinning of inner retinal layers inferotemporal to the fovea in the left eye, accompanied by a peripapillary hemorrhage on the right. Laboratory examinations confirmed positive serology results, with a TPPA titer of 1:1280. Sixth nerve palsy was also noted as a systemic symptom. Fundus fluorescein angiography depicted mild late disc hyperfluorescence and a small hyperfluorescent area corresponding to a plaque. The patient received dexamethasone 0.1% and cyclopentolate 1% as topical treatments and was subsequently referred to the infectious diseases department. During a follow-up in 2016, he was discharged with visual acuities of 20/400 and 20/100 in both eyes and an IOP of 15 mmHg in both eyes. In 2018, the patient experienced bilateral panuveitis. The follow-up continued, culminating in final visual acuities of 20/20 and 20/32 in the right and left eyes, respectively, with final IOP of 7 mmHg and 6 mmHg in the right and left eyes, respectively.

Case 3

**Figure 4:**
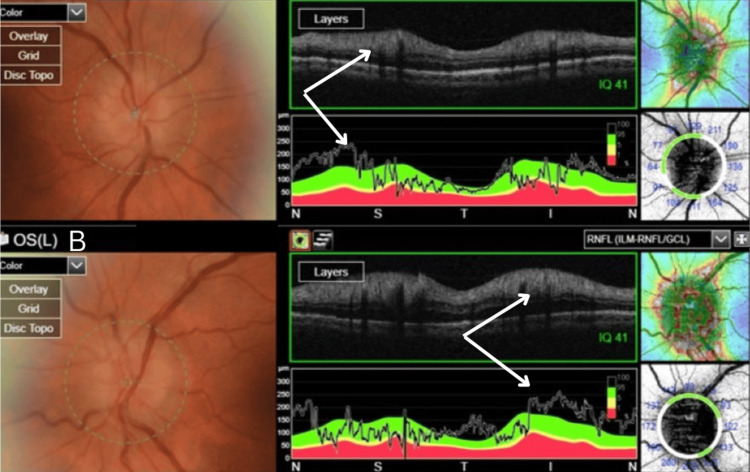
Bilateral OCT disc showing optic disc swelling secondary to optic neuritis/perineruitis OCT and RNFL analysis of both eyes. (A) OCT of the right eye showing optic disc swelling and RNFL thickening consistent with optic neuritis/perineuritis (arrows). (B) OCT of the left eye showing optic disc swelling and RNFL thickening consistent with optic neuritis/perineuritis (arrows). OCT: optical coherence tomography, RNFL: retinal nerve fiber layer

A 52-year-old male, known to be HIV positive, presented with symptoms suggestive of meningitis, including persistent buzzing in his ears, muffled hearing in the left ear, and blurred vision. Examination revealed bilateral optic disc swelling, as seen in Figure 4, and mild vitritis. Brain imaging showed subtle ventricular enlargement, while abdominal ultrasound indicated splenomegaly. Audiogram results revealed bilateral sensorineural hearing loss, primarily affecting high frequencies. The diagnosis of neurosyphilis was established, with the patient exhibiting 20/20 vision in both eyes and normal IOP. Laboratory tests confirmed positive syphilis serology, with a VDRL titer of 1:32 and a TPPA titer of 1:640. Bilateral hearing loss was also noted. Systemic treatment involved intravenous benzylpenicillin for 14 days, followed by a switch to intramuscular procaine penicillin from April 4 to September 4, along with a single dose of oral steroids. The patient was discharged and underwent follow-up in 2019, concluding with a final VA of 20/20 in both eyes.

Case 4

A 26-year-old man sought evaluation at the clinic after experiencing persistent floaters in his right eye for six weeks. On examination, he presented with extensive floaters and cobwebs in both eyes, accompanied by tunnel vision in the left eye and a sensation of throbbing. Clinical assessment revealed intermediate-mild vitritis and snowbanks, along with drusen in the macula of the right eye upon retinal imaging. The diagnosis of bilateral ocular syphilis was established. VA was limited to hand motion, with IOP measuring 11 mmHg in the right eye and 12 mmHg in the left eye. Systemically, he reported intermittent headaches. Laboratory tests demonstrated a TPPA titer exceeding 1:1280, a VDRL titer of 1/4, and positive syphilis serology. The treatment regimen included topical dexamethasone 0.1%, systemic methylprednisolone 1 g, followed by a two-week course of intravenous benzylpenicillin and a seven-day course of oral prednisolone. Follow-up visits ensued, culminating in the last one in 2020, during which a final VA of 20/25 in the right eye and 20/20 in the left eye was noted, with IOP of 14 mmHg in the right eye and 12 mmHg in the left eye.

Case 5

**Figure 5:**
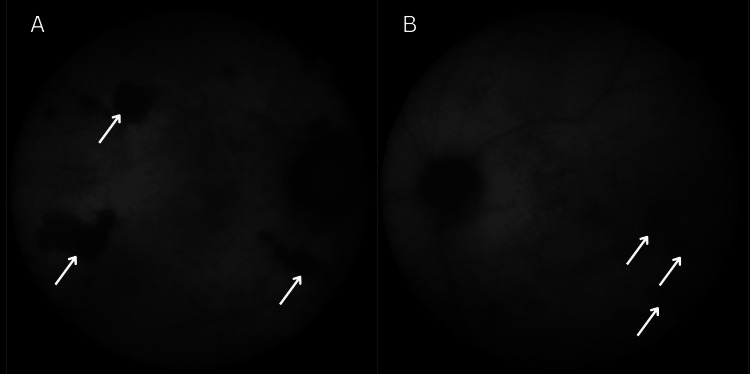
Bilateral FAF showing multiple hypofluorescence changes indication multifocal chorioretinal scar FAF images of both eyes. (A) Right eye. (B) Left eye. Both images show multiple areas of hypoautofluorescence scattered across the posterior pole and mid-periphery (arrows), with the right side greater than the left, corresponding to regions of RPE atrophy. FAF: fundus autofluorescence, RPE: retinal pigment epithelium

A 50-year-old man presented with a myriad of symptoms, including blurred vision, redness, painful watery eyes, and discomfort in the upper eyelids. He had a medical history notable for a distinctive retinal degeneration affecting both eyes alongside posterior uveitis. Retinal imaging revealed areas of atrophy, predominantly located inferiorly in the right eye, accompanied by vessel attenuation evident bilaterally. Generalized atrophy was noted on optical coherence tomography (OCT), complemented by patches of atrophy visible in fundus autofluorescence in both eyes, as seen in Figure 5. Following thorough evaluation, the diagnosis was confirmed as neurosyphilis, with a recorded VA of 20/50 in the right eye and 20/80 in the left eye, and IOP within the normal range. Subsequent admission to the infectious disease department yielded laboratory findings that revealed a VDRL titer of 1:8 and a TPPA titer of 1:1280. His last visit, dated May 20, 2019, showed marked improvement, with final visual acuities of 20/20 in the right eye and 20/32 in the left eye, alongside IOP measured at 6 mmHg in the right eye and 7 mmHg in the left eye.

Case 6

**Figure 6:**
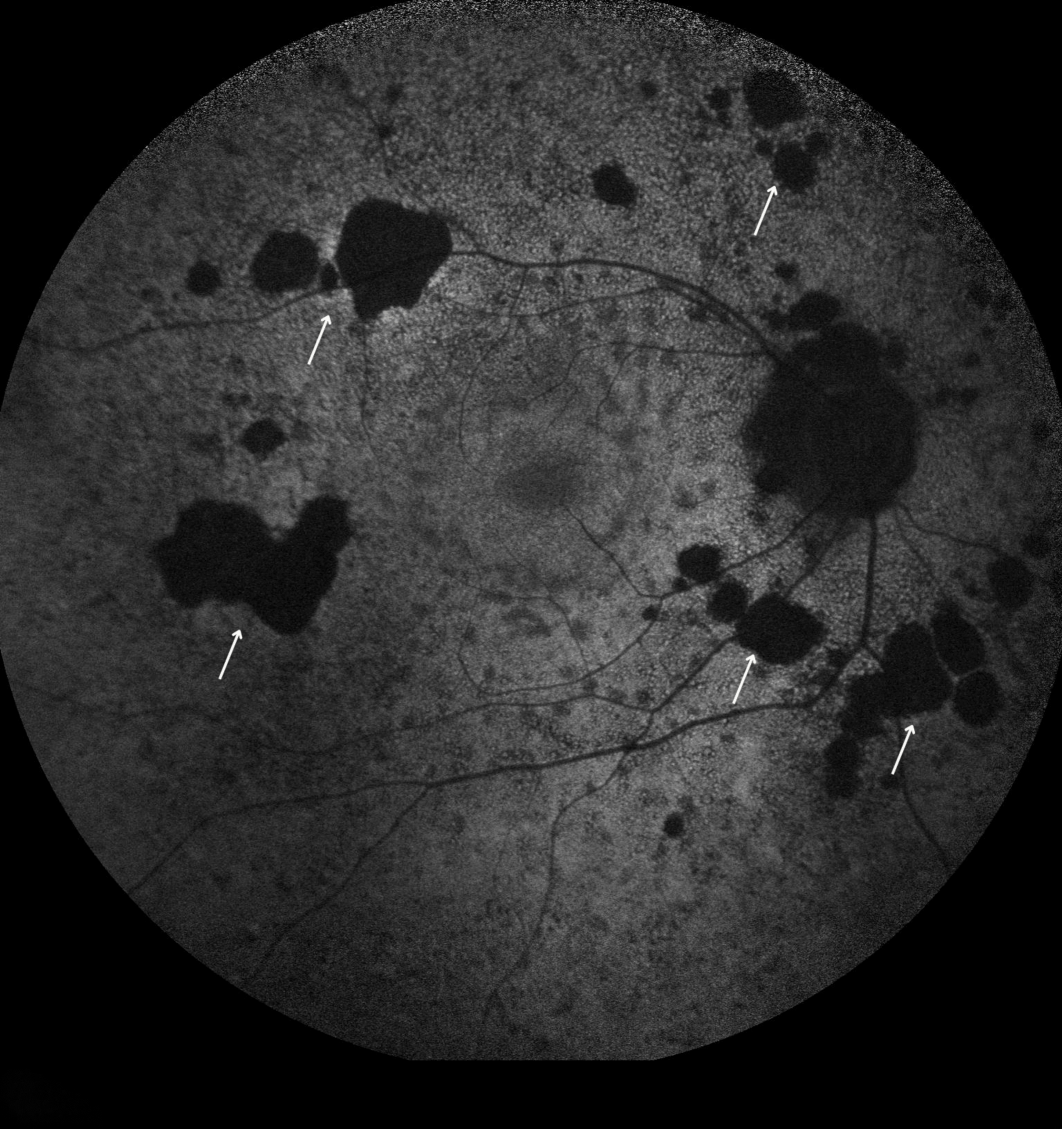
Right eye FAF showing multiple hypofluorescence changes indication multifocal chorioretinal scars FAF image of the right eye. The image reveals multiple large, well-demarcated areas of hypoautofluorescence (arrows) scattered throughout the posterior pole and mid-periphery, corresponding to regions of RPE atrophy. FAF: fundus autofluorescence, RPE: retinal pigment epithelium

A 57-year-old male presented with a sudden, painless loss of vision spanning six days. His medical history included previous episodes of blurred vision in the left eye, alongside a complex medical profile featuring hypertension, coronary heart disease, type 2 diabetes mellitus, Kawasaki disease, and a notable weight loss of 6.5 stones over 18 months. Clinically, the patient exhibited retinitis characterized by subretinal white lesions in the left eye. Retinal imaging revealed a subtle but significant loss in the inner segment/outer segment (IS/OS) layer, primarily inferotemporal to the fovea, extending superiorly in an arc-like pattern. A left-color photograph depicted a faint, pale area centrally around the fovea, corresponding to the loss and disruption of the IS/OS layer, accompanied by spiky projections from the retinal pigment epithelium. Autofluorescence in the right eye demonstrated superior hypo-autofluorescence as seen in Figure 6. In contrast, fluorescein angiography/indocyanine green imaging in the left eye indicated late leaks around the terminal vascular branches. The diagnosis confirmed ocular syphilis, linked with a VA of 20/100 in the right eye and 20/200 in the left, alongside IOP measured at 18 mmHg in the right eye and 14 mmHg in the left eye. The patient reported tingling sensations, tremors resembling a stocking-and-glove distribution pattern, occasional poor coordination, a painless glans ulcer treated as balanitis two years ago, and a history of painless oral ulcers associated with flu-like symptoms. Laboratory findings revealed positive syphilis serology, with a VDRL titer of 1:32 and a TPPA titer of 1:640. While topical treatments were not administered, systemic interventions were pursued, including intravenous ceftriaxone and oral prednisolone (60 mg). Notably, significant improvement was observed in the ellipsoid zone on OCT. Subsequently, the patient was referred to the infectious disease department, where, a few months later, the latest examination revealed a final VA of 20/25 in both eyes and IOP of 15 mmHg in the right eye and 16 mmHg in the left eye.

Case 7

In the following case, a 75-year-old male presented with blurred vision, red and painful watery eyes, and photophobia. He exhibited signs of suspected acute retinal necrosis, coupled with panuveitis. Retinal images revealed changes consistent with a "salt and pepper" appearance, along with evident perivenous sheathing, particularly in the retinal periphery. This was corroborated by widespread mottled hyper- and hypofluorescence, venous leakage, and signs of ischemia observed during fluorescein angiography of the right eye. Fundus examination of the left eye displayed significant retinal whitening and hemorrhages centered around the posterior pole. The diagnosis indicated bilateral ocular syphilis and left amblyopia. VA was measured at 20/250 in the right eye and 20/400 in the left eye, with an IOP of 10 mmHg in both eyes. Laboratory findings confirmed positive syphilis serology and Salisbury. Topical treatment was recommended, including sectoral panretinal photocoagulation for the left eye due to the presence of an ischemic area in the superonasal quadrant. The last examination, conducted a few months later, revealed a final VA of 20/400 in the right eye and hand motion in the left eye, with an IOP of 20 mmHg in the right eye and 16 mmHg in the left eye.

Case 8

A 32-year-old male presented to the clinic with complaints of blurred vision and a sudden-onset central scotoma persisting for over 10 days in his left eye, along with photopsia initially mistaken for Krill's disease. Bilateral syphilitic chorioretinitis was diagnosed, with the left eye exhibiting more severe symptoms than the right. VA was measured at 20/40 in the right eye and 20/80 in the left eye, with IOP of 14 mmHg and 15 mmHg, respectively. Retinal imaging revealed generalized vasculitis and disc leakage on fundus fluorescein angiography. Laboratory tests confirmed the diagnosis of bilateral ocular syphilis, with significantly elevated VDRL and TPPA titers of 1:512 and 1:1280, respectively. Treatment comprised intravenous penicillin and a three-day course of 500 mg methylprednisolone, following which the patient was admitted to the infectious disease department. At the year-end examination, mild focal hypopigmentary changes were noted. Final VA was recorded as 20/25 in both eyes, with IOP of 15 mmHg in the right eye and 16 mmHg in the left eye.

Case 9

A 46-year-old male presented to the eye casualty with eye pain, redness, photophobia, and blurred vision. Additional evidence of anterior uveitis, including superficial punctate keratitis, cells rated at 4+, Descemet's membrane folds, and perilimbal injection, was observed during examination. Retinal imaging, including Heidelberg OCT, fundus fluorescein angiography, indocyanine green angiography, and OPTOS widefield retinal photography, revealed no abnormalities. His VA was measured at 20/200 in one eye and 20/20 in the other, while the IOP was 8 mmHg in both eyes. Laboratory testing uncovered a TPPA titer of 1:1280, a VDRL titer of 1:64, and positive syphilis IgM, leading to the diagnosis of neurosyphilis. The patient reported intermittent headaches and presented with a maculopapular rash on the palms, trunk, legs, and penis, persisting for three to four weeks. Further, MRI findings indicated lesions causing seizures, for which the patient received treatment with carbamazepine and clobazam. Systemic treatments included a 10-day course of IV benzylpenicillin (initially IV ceftriaxone for five days), while topical treatments involved dexamethasone 0.1% and cyclopentolate 1%. Subsequently, the patient was admitted to the infectious disease unit. In 2023, during the last examination, the IOP was measured at 14 mmHg in the right eye and 15 mmHg in the left eye. The final unaided VA is 20/80 in the right eye and 20/25 in the left eye, respectively.

## Discussion

Ocular syphilis refers to the infection of the visual system resulting from the invasion of *T. pallidum* into the eyes, causing an inflammatory reaction. While ocular syphilis is considered rare, its prevalence rate has been steadily increasing over the past decade [7]. It can manifest six weeks or more after acquisition and may sometimes be the sole indication of systemic syphilis. Initially, ocular syphilis is uncommon, except for involvement of the eyelids and conjunctiva. However, in the second stage, ocular symptoms such as scleritis, keratitis, iris nodules, and iridocyclitis may become apparent [8]. Previous case studies have reported ocular symptoms associated with syphilis infection occurring in 0.6% to 2.7% of all cases [7,9-11]. In this particular case series, all nine confirmed cases of ocular syphilis presented with ocular manifestations.

According to Dombrowski et al., a review of 573 cases of syphilis in Washington revealed that 68 cases were suspected of having neurosyphilis, with 48 classified as early-stage syphilis and 20 as late- or unknown-stage syphilis. Of these, 3.5% exhibited both symptoms and objective confirmation of complicated syphilis, as evidenced by an abnormal ophthalmologic examination, while 7.9% reported changes in vision or hearing [10]. The study further noted that among confirmed cases of complex syphilis, symptoms of complicated syphilis were more frequently observed in patients with late- or unknown-stage syphilis (13.1%) compared to those with early syphilis (7.0%). Within the early syphilis group, symptoms of complicated syphilis were most prevalent during the secondary stage (10.3%), followed by the early latent (7.1%) and primary (0.9%) stages. Moreover, the prevalence of confirmed complex syphilis was higher in secondary (3.8%) and early latent (3.1%) stages compared to primary syphilis (0.9%) [10].

The hallmark symptoms of ocular syphilis often manifest as inflammation, such as anterior uveitis, intermediate uveitis, and posterior uveitis, potentially affecting either one or both eyes. Additional manifestations encompass complicated or severe inflammation such as syphilitic meningitis, retinal vasculitis, optic neuropathy, and interstitial keratitis. Uveitis associated with syphilis typically presents with eye redness, pain, and diminished VA [8,12]. In severe cases, hypopyon and posterior synechiae may develop as a result of inflammation. Intermediate uveitis related to syphilis is also rare and may manifest as peripheral retinal vasculitis. Our findings indicate that all nine cases were diagnosed with uveitis, either anterior, posterior, or panuveitis. VA among patients varied widely, ranging from normal to as low as light perception vision, with the majority exhibiting posterior segment abnormalities in imaging. Syphilis-related uveitis may lack characteristic ocular symptoms in its early stages, so the diagnostic approach plays a vital role in defining it. Determining the possible etiology of uveitis is essential to the diagnosis process. The findings showed that the two most common etiologies of anterior uveitis are herpetic and idiopathic (greater proportion), with other etiologies following. A significant portion of cases of intermediate uveitis are likewise idiopathic. Finally, in panuveitis, idiopathic uveitis affects more than one-third of cases and is followed in smaller percentages by sarcoidosis, ABD, and phacoanaphylactic uveitis [13].

When diagnosing a patient with ocular inflammation, syphilis-related uveitis should be considered [14]. Irreversible vision loss may result from inappropriate therapy and delayed diagnosis [15,16]. For each patient, a well-fitted approach comprising laboratory tests, biomarkers, and a large set of clinical signs (ocular or systemic), as well as data from ophthalmic and systemic imaging, would assist in the accurate differential diagnosis of uveitis and its severity, as well as determining how well the patient responds to treatment. Kalogeropoulos et al. reported diagnostic and therapeutic algorithms allowing the efficient management of uveitis [13]. Ocular and brain imaging were helpful in both determining the underlying cause of uveitis and tracking its healing process. The authors identified several abnormalities associated with uveitis, including retinal and choroidal lesions, vasculitis, optic nerve issues, retinal ischemia, and macular edema, which can be visualized using multimodal imaging and provide valuable information.

HIV coinfection modifies syphilis severity and raises the risk of syphilitic central nervous system involvement. In patients with syphilis and HIV infection who are not on antiretroviral medication, ocular involvement is more often bilateral and appears to involve the posterior segment more frequently [17]. Several publications, case reports, and case series in recent years have revealed the incidence of ocular syphilis in patients with HIV infection, showing that syphilitic eye involvement is more prevalent in the HIV population [18-22]. More severe forms of inflammation, such as necrotizing retinitis and posterior placoid chorioretinitis, bilateral necrotizing retinitis with serous retinal detachment, and chorioretinitis, are within the range of posterior segment morbidity. Scleritis likely attributable to syphilis was observed in a patient with HIV infection undergoing immunological reconstitution [23].

Kalogeropoulos et al. published a report of a case series of HIV-positive syphilitic uveitis (not only HIV+ were included in this one). Twelve individuals with active syphilitic uveitis who were HIV positive were gathered. Three of the five had a history of syphilis and had also received anti-syphilis medication. Some ocular symptoms were observed, including retinal hemorrhage, optic disc edema, macular edema, corneal epithelial defect, and complex cataract. As well, the literature suggested that a summary of 105 previously documented instances of HIV-positive syphilitic uveitis was found [22]. Symptoms included a drop in vision, redness, floating debris, headache, ocular pain (13%), and visual field abnormalities. The symptoms last for five to twelve months.

In another study, Balba et al. examined HIV patients on highly active antiretroviral therapy and discovered that three (9%) out of 33 syphilis patients presented with ocular symptoms. Six percent of HIV-positive syphilis patients had ocular disease, according to a previous study by Marra et al. [19,24]. In our case series, one patient experienced HIV coinfection with ocular syphilis and had symptoms of meningitis, tinnitus, muffled hearing, and blurred vision. He showed mild vitritis and optic disc swelling and was diagnosed with neurosyphilis.

Regarding the treatment of ocular syphilis, the gold standard is 14 days or more, if necessary, of penicillin G and ceftriaxone or doxycycline in patients with penicillin allergy [25]. Dosage and duration are dependent on the severity of the disease. In addition to systemic treatment, local drops have been found effective in reducing inflammation and slowing disease progression. The primary topical drops used are steroids to reduce inflammation. Adjuvant drops, such as lubricating drops and mydriatics, are used to alleviate ocular symptoms [25,26]. In our case series, most patients received systemic treatment with intravenous antibiotic benzylpenicillin, except for one who was administered intravenous ceftriaxone.

Additionally, systemic prednisolone was incorporated into the treatment plan for four patients. In a study conducted by Kalogeropoulos et al., three patients were prescribed topical steroids and cycloplegics to alleviate pain and prevent iris synechiae as part of their topical treatment regimen. Also, in patients with HIV-positive ocular syphilis, treatment with penicillin, ceftriaxone sodium, or penicillin with benzylpenicillin rather than benzylpenicillin alone was documented to improve corrected VA significantly [13,22]. Ocular syphilis patients may not respond to treatment. Even after receiving the proper antibiotic treatment, monitoring should continue to identify reinfection or recurrence.

Limitations of the study

This study has several limitations that should be considered when interpreting the results. First, its retrospective design may introduce selection bias and limit the completeness and accuracy of the collected data. The small sample size reduces the statistical power of the findings and restricts their generalizability to broader populations. Additionally, as a single-center study, the results may not be representative of other clinical settings or patient demographics. Variability in clinical documentation and follow-up duration further limits the ability to assess long-term outcomes and treatment efficacy. Finally, the absence of a control group makes it difficult to draw definitive conclusions about causality or the comparative effectiveness of different management strategies. A larger, prospective multicenter study would help validate these findings.

## Conclusions

Irrespective of the syphilis stage, ocular manifestations of syphilis may manifest with symptoms of vision impairment. They can also mimic other eye conditions and serve as the sole indicator of syphilis. Hence, even in cases where the medical history does not initially raise suspicion, it is imperative to rule out syphilis infection in all patients presenting with uveitis, optic neuritis, optic atrophy, sudden ocular muscle paresis, or loss of VA. Inadequate treatment and delayed diagnosis are often associated with lasting consequences for the affected individual. The consideration and exclusion of HIV infection, along with thorough investigation for syphilis, are crucial, particularly when ocular symptoms are present.
